# Establishment of a Non-Invasive Semi-Quantitative Bioluminescent Imaging Method for Monitoring of an Orthotopic Esophageal Cancer Mouse Model

**DOI:** 10.1371/journal.pone.0114562

**Published:** 2014-12-10

**Authors:** Shinji Kuroda, Tetsushi Kubota, Katsuyuki Aoyama, Satoru Kikuchi, Hiroshi Tazawa, Masahiko Nishizaki, Shunsuke Kagawa, Toshiyoshi Fujiwara

**Affiliations:** 1 Department of Gastroenterological Surgery, Okayama University Graduate School of Medicine, Dentistry and Pharmaceutical Sciences, Okayama, Japan; 2 Center for Innovative Clinical Medicine, Okayama University Hospital, Okayama, Japan; University of Pennsylvania, United States of America

## Abstract

Orthotopic models of various types of tumors are widely used in anti-tumor therapeutic experiments in preclinical studies. However, there are few ways to appropriately monitor therapeutic effect in orthotopic tumor models, especially for tumors invisible from the outside. In this study we aimed to establish a non-invasive semi-quantitative bioluminescent imaging method of monitoring an orthotopic esophageal cancer mouse model. We confirmed that the TE8 esophageal cancer cell line implanted orthotopically into the abdominal esophagus of *nu/nu* mice (n = 5) developed not only a main tumor at the implanted site, but also local lymph node metastases and peritoneal disseminations within 6 weeks after inoculation. We established a TE8 cell line that stably expressed the firefly luciferase gene (TE8-Luc). We showed that TE8-Luc cells implanted subcutaneously into *nu/nu* mice (n = 5) grew over time until 5 weeks after inoculation. Tumor volume was strongly correlated with luminescent intensity emitted from the tumor, which was quantified using the IVIS imaging system. We then showed that TE8-Luc cells implanted orthotopically into the mouse abdominal esophagus (n = 8) also formed a tumor and that the luminescent intensity of such a tumor, as detected by IVIS, increased over time until 7 weeks after inoculation and was therefore likely to reflect tumor progression. We therefore propose that this orthotopic esophageal cancer model, monitored using the non-invasive semi-quantitative IVIS imaging system, will be useful for in vivo therapeutic experiments against esophageal cancer. This experimental setting is expected to contribute to the development of novel therapeutic technologies for esophageal cancer in preclinical studies.

## Introduction

Esophageal cancer is one of the most common types of cancer in the world. Histologically, squamous cell carcinoma accounts for more than 90% of cancers in Japan, while adenocarcinoma accounts for more than half of cancers in the U.S. The prognosis of esophageal cancer is quite poor among cancers of the gastrointestinal tract, such as gastric cancer and colon cancer as esophageal cancers are prone to lymph node metastases and direct invasion to the surrounding organs. Despite progress in early diagnosis and in the development of surgical techniques and devices, as well as in chemotherapy, radiotherapy and their combination, esophageal cancer has not yet been satisfactorily overcome and this is a field in which new effective therapeutics are expected to be developed in the near future [1]–[3].

In the field of drug discovery, orthotopic tumor models are undoubtedly better models than subcutaneous models for evaluation of the therapeutic efficacy of new drugs because they better reflect disease progression of the original tumors [4], [5]. A variety of orthotopic tumor models have been used in animal studies including models of colon, pancreatic, liver, lung, prostate, renal cell, bladder, breast, melanoma and brain tumors [6]. However, there are only a few reports of orthotopic esophageal cancer models despite the urgent need of such a model. The lack of an orthotopic esophageal cancer model appears to be due to the problems of anatomical location and size of the esophagus in addition to the technical difficulty of establishing an orthotopic model.

The availability of a method for evaluating therapeutic efficacy is another issue when using an orthotopic model. Although survival rate or body weight has been commonly used for evaluation, these parameters do not directly reflect therapeutic efficacy because of the influence of a number of other factors on such efficacy. A few diagnostic modalities have been used as non-invasive methods for the monitoring of an orthotopic model, such as magnetic resonance imaging (MRI) [7], [8] and positron emission tomography (PET)/computed tomography (CT) [9], [10]. In addition, the use of green fluorescent protein (GFP)- [4], [11] and luciferase- [12], [13] expressing cell lines have provided an interesting approach to the development of a non-invasive imaging technique.

Here we report the establishment of an original orthotopic esophageal cancer model in mice, which developed local lymph node metastases and peritoneal disseminations in addition to the main tumor. Using this model we confirmed that the IVIS imaging system (Xenogen, Alameda, CA) was appropriate and useful as a non-invasive monitoring method for tumor progression in this model.

## Materials and Methods

The animal experimental protocol was approved by the Ethics Review Committee for Animal Experimentation of Okayama University, and all mice used in this study were anesthetized with ketamine/xylazine or isoflurane/oxygen for experiments and euthanized with cervical dislocation under anesthesia.

### Cell lines and cell cultures

The human esophageal squamous cell carcinoma line, TE8 (RIKEN BRC Cell Bank, Japan) was cultured in RPMI 1640 medium supplemented with 10% fetal calf serum (FCS), 100 units/ml penicillin and 100 µg/ml streptomycin. TE8 transfected with the firefly luciferase plasmid vector (pGL-Control-RSVneo) (kindly provided by Dr. Hiroyuki Mizuguchi, Osaka University, Osaka, Japan) and cloned by limiting dilution (TE8-Luc) was cultured as described for TE8 above but with the addition of 0.2 mg/ml Geneticin (G418) (Life Technologies, 10131-027) to the medium. The average level of luciferase activity per 5.0 × 10^4^ TE8-Luc cells was 1462 relative light units (RLU).

### Subcutaneous tumor model

TE8-Luc cells (2×10^6^ cells/mouse) were injected subcutaneously into the flank of five 5–6 week old female BALB/c *nu/nu* mice. The perpendicular diameter of each tumor was measured once a week until 5 weeks after tumor inoculation. Tumor volume was calculated using the following formula: tumor volume (mm^3^)  =  *a*×*b* ^2^×0.5, where *a* is the longest diameter, *b* is the shortest diameter, and 0.5 is a constant to calculate the volume of an ellipsoid.

### Orthotopic esophageal cancer model

Female BALB/c *nu/nu* mice (5–6 weeks old) were fixed in a supine position. A skin incision 7 to 10 mm long was made in the middle of the upper abdomen from the xiphoid process. The liver was raised up and the stomach was pulled down with tweezers so that the abdominal esophagus could be seen. A 1 ml syringe with a 31-gauge needle, in which TE8 or TE8-Luc cells were suspended in Matrigel at a concentration of 2×10^6^ cells/20 µl, was inserted into the anterior wall of the stomach and then moved subserosally toward the abdominal esophagus across the esophagastric junction. The cells were then slowly injected. Finally, the abdomen was closed with sutures (Movie S1).

Using this method, TE8 cells were orthotopically implanted into five BALB/c *nu/nu* mice. At 4 weeks after tumor inoculation, all five mice were operated on again to check tumor development, and then one of the five mice was sacrificed and its main tumor and a local lymph node were harvested for histological examination. At 6 weeks after inoculation, the other four mice were all sacrificed after re-laparotomy and observation.

TE8-Luc cells were implanted into the abdominal esophagus of eight BALB/c *nu/nu* mice using the same method. At 3 weeks after tumor inoculation, all the mice were operated on to check tumor development, and then five of the eight mice, in which tumor formation at the esophagus was observed, were monitored once a week using the IVIS imaging system described below until 7 weeks after tumor inoculation.

### IVIS imaging

Luciferin, the substrate of luciferase, was injected intraperitoneally into mice at a dose of 150 mg/kg body weight. The mice were then anesthetized and placed on the imaging stage of the IVIS apparatus in the abdominal (subcutaneous model) or the supine (orthotopic model) position. Images were collected every few minutes from 10 to 30 minutes after luciferin injection using the IVIS Imaging System (Xenogen, Alameda, CA), and photons emitted from the tumor and its surroundings were quantified using Living Image Software (Xenogen).

### Statistical analysis

Simple linear regression analysis was performed using Microsoft Excel to examine the correlation between tumor volume and luminescent intensity, and the coefficient of determination, R^2^, was calculated to assess the strength of the association.

## Results

**Figure 1:**
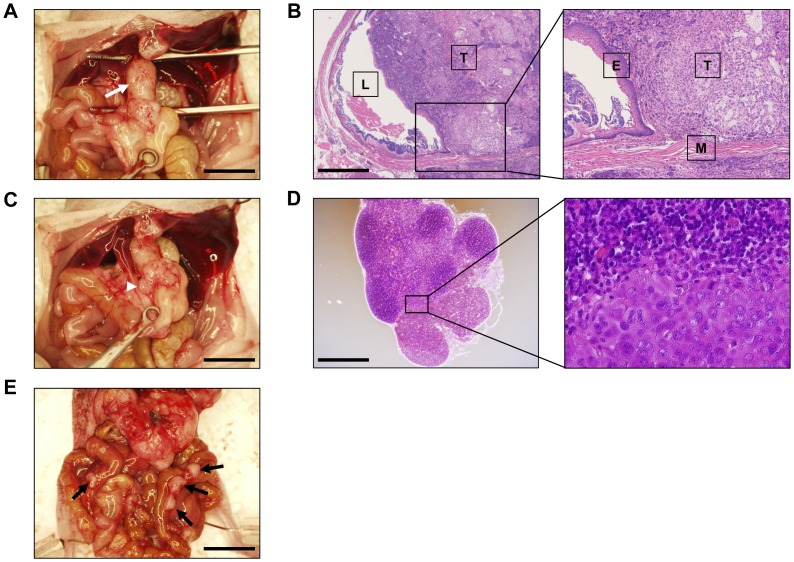
Establishment of a TE8 orthotopic esophageal cancer model. **A)** TE8 cells implanted into the abdominal esophagus formed a tumor at the implanted site (white arrow) at 4 weeks after tumor inoculation. **B)** H.E. staining of the paraffin-embedded tissue of the main tumor (A) showed that the tumor had spread into the submucosal space of the abdominal esophagus. (T: Tumor, L: Lumen, E: Epithelium layer, M: Muscle layer) **C)** TE8 cells implanted into the abdominal esophagus developed local lymph node metastases (white arrowhead) at 4 weeks after tumor inoculation. **D)** H.E. staining of the paraffin-embedded tissue of the local lymph node (C) histologically indicated lymph node metastasis. **E)** TE8 cells implanted orthotopically also developed peritoneal disseminations (black arrows) at 6 weeks after tumor inoculation. Scale bars; 2 mm (A, C, E), 500 µm (B, D)

TE8 cells injected into the subserosal space of the abdominal esophagus of BALB/c *nu/nu* mice formed a tumor (Fig. 1A) in four of the five mice and developed local lymph node metastases (Fig. 1C) in two of the five mice at 4 weeks after tumor inoculation. At 6 weeks after inoculation, another mouse had findings of peritoneal disseminations (Fig. 1E). Formation of a main tumor (Fig. 1B) and metastases to local lymph nodes (Fig. 1D) were also confirmed by histological findings. The main tumor grew in the submucosal space despite being injected into the subserosal space, whereas it did not invade the mucosal layer.

**Figure 2:**
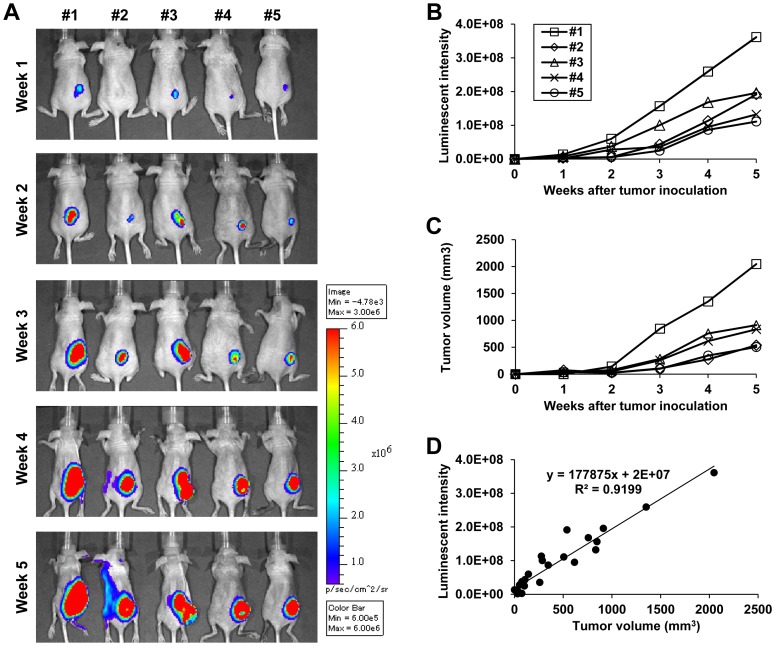
Assessment of the IVIS imaging system as a method for monitoring tumor growth in a TE8-Luc subcutaneous tumor model. **A)** IVIS images were obtained once a week until 5 weeks after subcutaneous inoculation of TE8-Luc cells. **B)** Luminescent intensity of photons emitted from each tumor in the images in (A) was quantified. Mouse numbering corresponds to the numbering in (A). **C)** Tumor volume was also calculated once a week. **D)** Correlation between luminescent intensity emitted from each tumor and tumor volume was statistically evaluated using the data of (B) and (C).

We next generated TE8-Luc cells, which stably express the firefly luciferase gene, and subcutaneously implanted these cells into BALB/c *nu/nu* mice. The luminescence of these cells was monitored with the IVIS imaging system to determine if tumor volume correlated with luminescent intensity. TE8-Luc cells formed a tumor in all five mice. As the tumors grew, luminescent intensity increased over time as assessed both visually (Fig. 2A) and quantitatively (Fig. 2B) (Fig. 2C). Tumor volume and luminescent intensity were strongly correlated with each other (R^2^ = 0.9199) (Fig. 2D), indicating that measurement of luminescent intensity is appropriate for evaluation of tumor progression.

**Figure 3:**
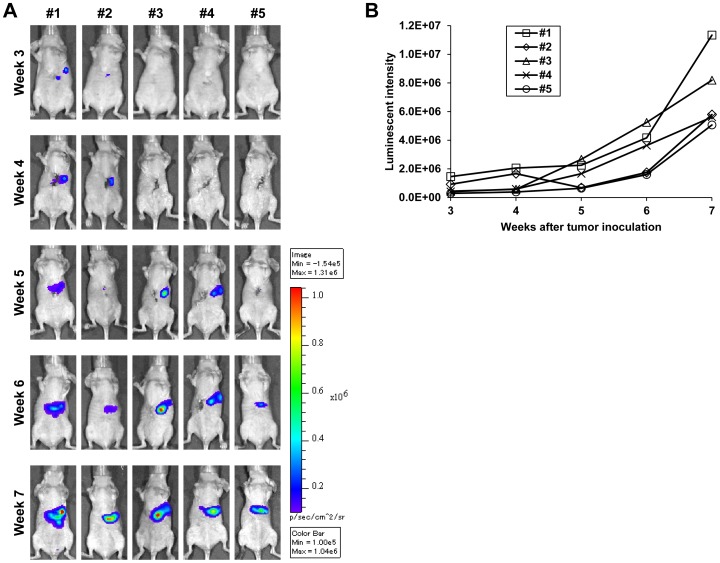
Establishment of a TE8-Luc orthotopic esophageal cancer model and a non-invasive semi-quantitative monitoring method using the IVIS imaging system. **A)** IVIS images were obtained once a week from 3 to 7 weeks after TE8-Luc orthotopic tumor inoculation into the abdominal esophagus. **B)** Luminescent intensity of photons emitted from each tumor and its surroundings in the images in (A) was quantified. Mouse numbering corresponds to the numbering in (A).

Finally, we determined if TE8-Luc cells could form an orthotopic tumor that grew over time. Five of eight BALB/c *nu/nu* mice in which TE8-Luc cells were implanted orthotopically into the abdominal esophagus developed a tumor at the implanted site at 3 weeks after tumor inoculation, which was confirmed by re-laparotomy. These five mice were monitored by IVIS once a week, and photons emitted from the tumor and its surroundings increased over time as assessed both visually (Fig. 3A) and quantitatively (Fig. 3B).

## Discussion

Furihata et al. [14] were the first to report establishment of an orthotopic esophageal inoculation model in mice in 2001, in which they opened the anterior wall of the stomach and injected tumor cells into the submucosal space of the abdominal esophagus directly after laparotomy. In contrast, we injected cells into the subserosal space of the abdominal esophagus without opening the stomach. Although their method is theoretically a more ideal method than ours, our method is simpler and is still an acceptable method because tumors formed using our method grew and spread in the submucosal space despite being injected into the subserosal space as the histological findings in Fig. 1C show. Two of five of our model mice developed local lymph node metastases and one mouse exhibited peritoneal disseminations. Obvious metastases to liver, lung or mediastinal lymph nodes were not found. In order to generate a more invasive orthotopic esophageal cancer model, it may be necessary to use more invasive cells [15] or to use clinical tumor samples that have high invasive potential [4].

Ohara et al. [16] established an interesting orthotopic mouse model of cervical and thoracic esophageal cancer by injecting tumor cells into the esophageal lumen through the mouth. While this method seems simple in that no surgical technique is required, it may be difficult to judge if tumor cells are accurately injected into the esophagus because the procedure is inevitably performed blindly. However, this method may have an advantage over our method in that it causes poor oral intake, which better reflects progression of the original tumor. In our study, body weight did not decrease at all until just before death, despite extensive tumor growth and spread (data not shown).

Quatromoni et al. [17] recently reported effective adenoviral-based immunotherapy against esophageal cancer, in which an orthotopic esophageal cancer mouse model established at the gastroesophageal junction using the same technique as we showed in the movie was used. In this model, tumors grew over time confined to the tumor-injection site without direct invasion of the esophageal mucosa. However, tumors did not cause obstruction of the esophageal lumen and no mice had evidence of dysphagia and significant weight loss. These characteristics are all similar with those we showed in our study.

In contrast, Gros et al. [18], [19] established an orthotopic esophageal cancer mouse model using a highly aggressive esophageal cancer cell line in the same way as ours, in which they detected metastatic spread to the liver and lungs as well as to the lymph nodes using high resolution imaging with GFP and by MRI while we were not able to detect metastases to the liver or other organs by luciferase imaging in our study. These reports from Quatromoni et al. and Gros et al., including our study, suggest that the orthotopic esophageal cancer model established at the gastroesophageal junction and its establishment method is universal and reproductive, and that this model can cause tumor progression, which is close to actual clinical practice, such as metastases to the lymph nodes, the peritoneum and the liver or other organs in addition to local tumor growth.

Unlike subcutaneous models, it is difficult to accurately analyze tumor progression in orthotopic tumor models. Although survival rate and body weight are commonly used as evaluation methods in orthotopic studies, these parameters do not directly reflect therapeutic efficacy because many other factors also influence the results. The use of tumor cells marked with luciferase reporter gene and bioluminescent imaging, which is currently a well-used method in research field, allows us to monitor tumor growth non-invasively in vivo, especially in deep tissues[13]. Therefore, we originally established TE8-Luc cells that were stably-transfected with the firefly luciferase gene and used for non-invasive monitoring.

In our study, using the IVIS imaging system, the luminescent intensity of photons emitted from TE8-Luc subcutaneous tumors strongly correlated with tumor growth. Based on this fact, it is therefore likely that an increase in luminescent intensity in an orthotopic model over time accurately reflects tumor progression. Indeed, in a previous study in which we quantitatively monitored therapeutic efficacy in this orthotopic model using the IVIS imaging system, the change in luminescent intensity did appear to accurately reflect therapeutic efficacy [20]. Therefore, the method that we describe in the present study for establishment of an orthotopic esophageal cancer model in mice and for non-invasive monitoring with the IVIS imaging system is a very useful and practicable method, and is expected to contribute to the development of novel therapeutic technologies for esophageal cancer.

## Supporting Information

Movie S1 **How to make an orthotopic esophageal cancer mouse model.**
(MPG)
